# Methodological Issues in the Clinical Validation of Biomarkers for Alzheimer’s Disease: The Paradigmatic Example of CSF

**DOI:** 10.3389/fnagi.2019.00282

**Published:** 2019-10-17

**Authors:** Marco Canevelli, Ilaria Bacigalupo, Giuseppe Gervasi, Eleonora Lacorte, Marco Massari, Flavia Mayer, Nicola Vanacore, Matteo Cesari

**Affiliations:** ^1^Department of Human Neuroscience, Sapienza University, Rome, Italy; ^2^National Center for Disease Prevention and Health Promotion, National Institute of Health, Rome, Italy; ^3^National Center for Drug Research and Evaluation, National Institute of Health, Rome, Italy; ^4^Fondazione IRCCS Ca’ Granda Ospedale Maggiore Policlinico, Milan, Italy; ^5^Geriatric Unit, Department of Clinical Sciences and Community Health, University of Milan, Milan, Italy

**Keywords:** biomarkers, Alzheimer’s disease, validation, diagnostic research, epidemiology, mild cognitive impairment

## Abstract

The use of biomarkers is profoundly transforming medical research and practice. Their adoption has triggered major advancements in the field of Alzheimer’s disease (AD) over the past years. For instance, the analysis of the cerebrospinal fluid (CSF) and neuroimaging changes indicative of neuronal loss and amyloid deposition has led to the understanding that AD is characterized by a long preclinical phase. It is also supporting the transition towards a biology-grounded framework and definition of the disease. Nevertheless, though sufficient evidence exists about the *analytical validity* (i.e., accuracy, reliability, and reproducibility) of the candidate AD biomarkers, their *clinical validity* (i.e., how well the test measures the clinical features, and the disease or treatment outcomes) and *clinical utility* (i.e., if and how the test improves the patient’s outcomes, confirms/changes the diagnosis, identifies at-risk individuals, influences therapeutic choices) have not been fully proven. In the present review, some of the methodological issues and challenges that should be addressed in order to better appreciate the potential benefits and limitations of AD biomarkers are discussed. The ultimate goal is to stimulate a constructive discussion aimed at filling the existing gaps and more precisely defining the directions of future research. Specifically, four main aspects of the clinical validation process are addressed and applied to the most relevant CSF biomarkers: (1) the definition of reference values; (2) the identification of reference standards for the disease of interest (i.e., AD); (3) the inclusion within the diagnostic process; and (4) the statistical process supporting the whole framework.

## Introduction

A biomarker is defined as a characteristic that is objectively measured and evaluated as an indicator of normal biological processes, pathogenic processes, or pharmacological responses to a therapeutic intervention (Biomarkers Definitions Working Group, 2001). The use of biomarkers is profoundly transforming medical research and practice (the so called “biomarker revolution”; Schisterman and Albert, 2012). In fact, they may: (1) support the identification of pathophysiological processes causing or contributing to diseases; (2) define and predict the individual’s health trajectories and clinical outcomes; and (3) help in selecting interventions and monitoring the response to treatments. Thus, they play a relevant role within the promise of precision medicine approaches where medical choices are driven by individually targeted genetic and biological profiles (Jameson and Longo, 2015).

Biomarkers are particularly relevant in the study of pathological conditions affecting the central nervous system (CNS), considering that brain tissue is not readily accessible for diagnostic or research purposes. Specifically, their adoption has triggered major advancements in the field of Alzheimer’s disease (AD) over the past years. For instance, the analysis of the cerebrospinal fluid (CSF) and neuroimaging abnormalities indicative of neuronal loss and protein deposition has led to the understanding that AD is characterized by a long preclinical phase (Jack et al., 2013). This finding has been responsible for opening new perspectives in researching novel preventive/therapeutic strategies. It has also supported the transition towards a biology-grounded framework and definition of the disease (Jack et al., 2018). Furthermore, the use of these markers, when adopted as surrogate measures of AD in animal models, has contributed in accelerating the development of possible disease-modifying treatments (Cummings et al., 2018).

To date, although increasingly adopted in specialized clinical settings (Frisoni et al., 2017), the use of biomarkers to detect AD is still recommended only for research purposes and in selected atypical cases (McKhann et al., 2011; Dubois et al., 2014; Jack et al., 2018). Their adoption in the routine clinical practice remains controversial as confirmed by different systematic reviews and meta-analyses reaching heterogeneous results on the topic (Noel-Storr et al., 2013; Olsson et al., 2016; Ritchie et al., 2017). In particular, though sufficient (albeit inconclusive) evidence exists about the *analytical validity* (i.e., is the test accurate, reliable, and reproducible?) of the proposed AD biomarkers (Hansson et al., 2018; Lewczuk et al., 2018), their *clinical validity* (i.e., how well the test measures the clinical features, and the disease or treatment outcomes) and *clinical utility* (i.e., if and how the test improves the patient’s outcomes, confirms/defines the diagnosis, identifies at-risk individuals, influences therapeutic choices) have not yet been fully proven (Frisoni et al., 2017; Kraus, 2018).

In the present article, we discuss some of the methodological issues and challenges that should be addressed in order to better assess the potential benefits and limitations of AD biomarkers. Without the intent of underestimating what has been done over the years in the field, the ultimate goal of the present article is to stimulate a constructive discussion aimed at filling the existing gaps and more precisely defining the directions of future research. The work is structured around four main aspects to be considered when adopting a biomarker in clinical practice: (1) the definition of reference values; (2) the identification of reference standards specific for the disease of interest (i.e., AD); (3) the proper inclusion and contextualization within the diagnostic process; and (4) the statistical process supporting the whole framework. In particular, these points will be addressed with regard to the most relevant CSF biomarkers.

## Definition of Reference Values

The validation of a candidate biomarker should follow two preliminary steps: (1) the assessment of its distribution in healthy people; and (2) the definition of the index test reference values (e.g., those included between the 2.5th and 97.5th percentile of the distribution, or within the interval of the mean ±1.96 standard deviations in case of symmetric distribution). The impact of common sociodemographic characteristics (e.g., age, sex, race/ethnicity) on the identified normal and abnormal values should also be considered (Sackett and Haynes, 2002; Haynes and You, 2009; Colli et al., 2014). It should be underlined, within this framework, how challenging (or even arbitrary) the selection of the reference group (i.e., healthy controls) to be used to define the 95% range of reference values might be.

To assess the methodology of the studies providing reference intervals for possible CSF biomarkers [i.e., amyloid peptides Aβ1–42 (Aβ42), total tau (T-tau), and 181-phospo-tau (P-tau)] in AD, we retrieved all available literature published up to May 2019. To this purpose, we performed a structured search on PubMed using the following search terms: (Aβ* OR A-β* OR A-beta* OR abeta* OR AB-42 OR *tau) AND (CSF OR liquor OR cerebrospinal OR cerebro-spinal) AND [(population* OR reference* OR normative*) AND (value* OR limit*)] AND (healthy OR normal OR normality OR average OR “general population”). The search strategy led to the identification of 155 abstracts. The full-texts of six selected studies were retrieved and assessed for inclusion based on the following predefined inclusion/exclusion criteria: being published in English; having sample size >50 subjects; defining as explicit aim the identification of reference intervals or limits for the considered biomarkers. Only two studies were included based on their pertinence with and relevance to the topic of interest (Sjögren et al., 2001; Burkhard et al., 2004). As reported in Table 1, the two included studies showed a high heterogeneity in how both methods and results were reported, thus limiting their hypothetical summarization. Both studies investigated the CSF dosage of Aβ42 and T-tau in hospital-based samples of subjects with a wide spectrum of age (i.e., ranging from less than 30 years to even more than 90 years). The studies adopted the 10th fractile (or percentile) to calculate the reference limit for Aβ42 and the 90th fractile (or percentile) to define the reference limit for T-tau. Important differences were observed for what concerns the age distribution and sex composition of the enrolled study samples. Although the inconsistencies in the reporting of results (e.g., different stratification for age groups) preclude the possibility of a direct comparison of the findings, a relevant discrepancy in the identified reference limits was evident in the two studies (e.g., for Aβ42: 150 ng/L vs. 500 ng/L, respectively). Finally, none of them assessed the role of individual characteristics (e.g., race and genetics) that could potentially affect results and conclusions.

**Table 1:** Studies reporting reference limits for CSF Aβ42 and T-tau in healthy people.

Author	Setting	Sample size	Age	Sex	Diagnostic criteria for healthy individuals	Statistics	Reference limits
Sjögren et al. (2001)	Norwegian University Norwegian Hospital	231 (total) 175 (age >50 years)	61.3 ± 18 (mean age ± SD) 21–93 (range)	177 F 54 M	No symptoms or signs of psychiatric or neurological disorders: People aged ≥60 years: MMSE score = 28–30 People aged <60 years: no criteria listed	>0.10 fractile (i.e., 10th percentile) for Aβ42 <0.9 fractile (i.e., 90th percentile) for tau	**Aβ42** 500 ng/L (not age dependent) **T-tau** 300 ng/L (for age range 21–50) 450 ng/L (for age range 51–70) 500 ng/L (for age range ≥71)
Burkhard et al. (2004)	University Hospital of Geneve	105 (total) 82 (age >50 years)	69, 56–78 (median, interquartile range) 29–96 (range)	61 F 44 M	Historical referral of absence of any CNS or PNS condition, or psychiatric condition, or chronic systemic illness possibly modifying the CSF proteins Face-to-face questionnaire covering the most common neurological symptoms Intake of medication potentially interfering with brain functions Neuropathological findings confirming the absence of any dementia (for 10 autopsy proven cases)	>10th percentile for Aβ42 <90th percentile for tau	**Aβ42** 150 ng/L, 90% Confidence Interval 90–172 ng/L (for age >49 years) **T-tau** 385 ng/L, 90% Confidence Interval 332–451 ng/L (for age >49 years)

## Defining Diagnostic Reference Standards for AD

The clinical validation of AD biomarkers is complicated by the lack of a unique diagnostic reference (Noel-Storr et al., 2013). Furthermore, the biological and clinical approaches to the diagnosis of AD have some relevant limitations. Neuropathology has traditionally been considered as the gold standard for the evaluation and judgment of clinical manifestations (McKhann et al., 1984). Nevertheless, its large-scale implementation is hampered by the difficulty of obtaining samples. However, the neuropathological characteristics of AD have a weak correlation with its phenotypic and clinical expression. In fact, it is well established that many individuals showing a high burden of AD pathology do not exhibit any clinical signs of the disease, whereas others with a limited amount of neuropathological changes had developed overt AD in life (Wallace et al., 2019). Beyond the absence of clear evidence supporting their causal role, some of the biological processes resulting in the AD neuropathological hallmarks (e.g., amyloid deposition) may have different pathogenic implications (Espay et al., 2019). They may, in fact, alternatively contribute to and accelerate neurodegeneration, represent epiphenomena, or even constitute compensatory mechanisms to molecular/cellular stress (Espay et al., 2019). Moreover, different latent factors, such as the individual’s frailty status, may moderate the relationship between AD pathology and dementia (Wallace et al., 2019). Finally, most of dementia cases (including AD dementia) are underlined by a mixed neuropathology (Boyle et al., 2018).

On the other hand, the adoption of clinical standards can be itself prevented by several obstacles. Logically, the cross-sectional validation of biomarkers against clinical criteria cannot result in an optimal diagnostic accuracy (Noel-Storr et al., 2013). Therefore, their use as prognostic markers, using longitudinal reference standards such as the conversion from MCI to AD dementia, are being increasingly considered for this purpose (Ritchie et al., 2014). However, the marked heterogeneity of these clinical outcomes may strongly confound their performance. For instance, the phenomenon of MCI conversion may occur in extremely variable times and ways, and be potentially affected by several additional, interacting factors (Grande et al., 2014). Moreover, it has been observed that a sizeable proportion of subjects with MCI shows a normalization of neuropsychological tests over time (Canevelli et al., 2016). Some subjects may follow even more complex clinical trajectories, by, for example, first reverting to normal cognition and subsequently progressing to dementia (Roberts et al., 2014). Theoretically, such a potential for multiple evolutions of MCI, shared by most of the risk conditions (Canevelli et al., 2017), implies the need to overcome the adoption of “classic” dichotomous outcomes (i.e., normal vs. pathological) preferring endpoints including at least 3 levels (i.e., improvement vs. stability vs. worsening). In other words, biomarkers could potentially support the identification not only of those subjects progressing to dementia, but also of those individuals showing an “inverse” trajectory towards normality. In this framework, the possibility of combining different biomarkers (or sets of biomarkers) should be considered with the objective of detecting the risk of decline as well as the possibility of restoration of a normal status.

## The Architecture of the Diagnostic Process

The actual validity and utility of a diagnostic test (e.g., a biomarker) can be summarized in a multistep process that should answer some crucial diagnostic questions, included in five iterative phases (Table 2; Sackett and Haynes, 2002; Haynes and You, 2009).

**Table 2:** The diagnostic research questions.

**Phase I: Do the test results in patients with the target disorder differ from those in normal people?**
This preliminary phase is important to provide novel insights on the pathophysiological mechanisms of the disease. It can be addressed by conducting cross-sectional studies confronting a convenience group of subjects known to have the disease and a group of people definitely known to not have it.∣rule
**Phase II: Are patients with certain test results more likely to have the target disorder than patients with other test results?**
The answer to this question can be derived by classic 2 × 2 contingency tables (or Error Matrices). The accuracy of the test (in terms of its results or cut-points) at distinguishing patients with the disease from normal controls is expressed by means of sensitivity, specificity, positive and negative predictive values and likelihood ratios∣rule
**Phase III: Does the test result distinguish patients with and without the target disorder among patients in whom it is clinically reasonable to suspect that the disease is present?**
Differently from the previous phase, the accuracy of the test is here explored in a “real world” scenario of routine clinical practice, that is among subjects whose clinical status is not already established (e.g., subjects referred from their general practitioners to specialist services for a clinical suspicion). Participants should, blindly, be assessed with both the test and what is considered as the diagnostic reference standard (ideally a gold standard).∣rule
**Phase IV: Do patients who undergo this diagnostic test have better health outcomes than similar patients who are not tested?**
This question strongly deals with the clinical utility of the test and concerns the health outcomes following the diagnostic/therapeutic choices resulting from the test findings. Ideally, such information could be obtained by the follow-up of subjects randomized to perform the test or not to perform it.
**Phase V: Does the use of the diagnostic test lead to better health outcomes at acceptable costs?**
This question refers to the cost-effectiveness (the so-called “value-for-money”) of the index test and can be answered by randomized controlled trials.

*Adapted from Haynes and You (2009) and Sackett and Haynes (2002)*.

In the AD literature, a relevant number of Phase I and Phase II studies has indicated that the CSF levels of biomarkers reflecting amyloid deposition (i.e., Aβ42) and neurodegeneration (i.e., T-tau and P-tau) are significantly different between subjects diagnosed with AD and to normal controls. In this context, a recent meta-analysis of 231 studies enrolling a total of 15,699 patients with AD and 13,018 controls reported an estimate of the following AD-to-control ratios: Aβ42 (average ratio 0.56, 95% CI 0.55–0.58, *p* < 0.0001), T-tau (2.54, 2.44–2.64, *p* < 0.0001), and P-tau (1.88, 1.79–1.97, *p* < 0.0001; Olsson et al., 2016). These biomarkers could also help in distinguishing those subjects with mild cognitive impairment (MCI) that will convert to dementia from non-converting subjects (Ritchie et al., 2017). Specifically, according to a recent Cochrane systematic review (Ritchie et al., 2017), the observed accuracy ranges of CSF biomarkers in predicting the conversion from MCI to AD dementia are:

- T-tau: sensitivity: 51%–90%; specificity: 48%–88%;
- P-tau: sensitivity: 40%–100%; specificity: 22%–86%;
- P-tau/Aβ42 ratio: sensitivity: 80%–96%; specificity: 33%–95%.

Such wide variability can be attributed to relevant discrepancies in the adopted reference standards, in the source of recruitment and sampling of participants, and in the index test methodology across the retained studies. It is to be noted that most of these results were obtained in research settings, evaluating highly selected patients in whom the presence of the target disease had already been ascertained under ideal/almost utopic circumstances (e.g., by expert clinicians with the best available equipment, adopting the same reference standard for those with and without AD). These samples are unlikely to represent the overall population of patients with AD under multiple socio-demographic and clinical aspects. Therefore, it seems reasonable to expect these same biomarkers to yield different results when transferred from the research to the clinical setting (Dyer et al., 2016; Frisoni et al., 2017). To date, only few studies have provided realistic information on the validity of AD biomarkers in the “real world” (thus answering pragmatic Phase III questions). As expected, a lower accuracy in the discrimination of patients with and without AD was observed in these works (Mattsson et al., 2009; Tariciotti et al., 2018). Moreover, to our knowledge, no Phase IV and V evidence are available in this field of AD research. In other words, no study has yet robustly explored how the use of biomarkers can actually affect health outcomes (e.g., mortality, disability, response to treatment; Frisoni et al., 2017) nor their cost-effectiveness.

## Statistical Approaches Across the Diagnostic Research Process

According to the previously discussed phases, different statistical approaches are required in each sequential step (Moons et al., 2012a,b; Collins et al., 2015). Phase I is exploratory by nature and is typically based on null hypothesis significance testing focused on isolating variables deemed individually relevant according to the *P*-value. The statistical methods for investigating Phase II and III questions belong to the field of prediction models (both diagnostic and prognostic) that typically focus on identifying sets of variables that can accurately predict the outcomes of interest. Considering the wide range of options and the differing perspectives of researchers, clinicians and public health decision makers, it is crucial to be aware about the trade-off between *model transparency* (allowing for easy interpretability and transparent scientific understanding) and *model complexity* (maximizing the predictive power through very sophisticated predictions that may often appear as an Opaque Black Box; Bzdok and Ioannidis, 2019). To this purpose, simple univariable classifications where Error Matrices (i.e., 2 × 2 contingency tables that report the number of *false positives*, *false negatives*, *true positives*, and *true negatives*) are derived by predefined cut-off values of single biomarkers as well as long-trusted multivariable statistical methods (e.g., Logistic and Cox Regression models) still remain the most suitable tools in the box. Regarding the Error Matrix and its derived measures (Akobeng, 2007), the Positive Predictive Value (PPV) and the Likelihood Ratio (LR) should always be preferred in prediction studies. In fact, Sensitivity and Specificity are indicative of the accuracy of a test (i.e., the biomarker), thus they are mostly useful for comparing the performance of different ones (with the possibility of combining two single tests in “OR”/“AND” modality to enhance the overall sensitivity/specificity; Sackett et al., 1985). The PPV and LR are, instead, informative about the single, specific individual. The PPV measures the individual probability to develop (or to have) the disease if the test is positive. The LR expresses the probability that the test is positive (or negative) in people with the disease compared to the probability that it is positive (or negative) in healthy people. It thus allows to simply update the pre-test probability of having the disease (based on the individual’s characteristics and clinical history) to the post-test probability (given the test results) according to its direction and magnitude (Table 3; Jaeschke et al., 1994; Kent and Hancock, 2016). Candidate CSF biomarkers for AD have so far shown small to minimum LR values (i.e., LR+ 2.72, LR− 0.32 at the median specificity of 72% for T-tau; LR+ 1.55, LR− 0.39 at the median specificity of 47.5% for P-tau; Ritchie et al., 2017).

**Table 3:** Definition and interpretation of the Likelihood Ratio (LR).

LR values	Change from pre-test to post-test probability	Results
>10 or <0.1	Large	Conclusive
5–10 or 0.1–0.2	Moderate	Moderately important
2–5 or 0.2–0.5	Small	Sometimes important
1–2 or 0.5–1	Small/minimum	Rarely important
1	None	Not important/useless

*Adapted from Jaeschke et al. (1994)*.

The predictive performance of a model is usually measured using discrimination measures (such as c-index that is equal to the area under the Receiver Operating Curve) and calibration plots. These measures can be inflated in the data sample from which they are derived when compared to new but comparable data samples (overfitting). K-fold cross-validation and bootstrap are the preferred internal validation techniques to evaluate a potential overfitting. However, external validation is still necessary to guarantee the generalizability of the model in the real word setting (Phase III). Finally, the appropriate reporting, communication and use of the resulting model are crucial. Therefore, the output of the predictive model (in terms of coefficient estimates, standard error and confidence intervals) can be combined to graphic tools, such as nomograms, thus easily allowing to obtain the final outcome probability for a new patient based on his/her profile of predictive variables. This graphical approach, although not widely used in the field of AD (Jang et al., 2017), may have important practical implications in the clinical and regulatory setting (e.g., patient’s counseling, risk stratification, elaboration of guidelines, drug reimbursement). Phase IV studies, while sharing inferential testing tools that are similar to those used in Phase I, are usually framed within an evidence-based decision-making context where the statistical methods are derived from the domain of well-controlled experimental study design (typically a randomized clinical trial). Phase V studies, instead, focus on the evaluation of the most effective or cost-effective diagnostic strategies through specific cost-effectiveness analysis.

## Conclusion

Overall, various methodological issues remain to be addressed in order to perform an adequate and complete clinical validation of candidate CSF biomarkers for AD. First, studies reporting the distribution of biomarkers in normal/healthy subjects and their variability according to major sociodemographic and clinical attributes are still lacking. In this regard, significant sex and race disparities for Aβ42 and tau levels have recently been reported both in healthy subjects and in patients with AD (Koran et al., 2017; Morris et al., 2019). Second, there is no conclusive agreement on the most appropriate reference standard for AD (e.g., clinical vs. biological) to be adopted to test the performance of new biomarkers. Third, no biomarker has yet consistently gone through all the phases that compose the architecture of diagnostic research. In particular, their actual impact on “hard” health outcomes and their cost-effectiveness has to be clarified. Similar conclusions have been reached by Mattsson et al. (2017) who have adopted an alternative model for developing the framework concerning AD biomarkers. Their approach, borrowed from oncology and structured around the natural history of the disease, should be regarded as complimentary to that adopted in the present work, essentially based on the methodological validation of biomarkers from the lens of clinical epidemiology. It is also crucial that, in each phase, the scientific contributions meet the highest quality standards. To this end, the widespread application of the checklist on reporting standards in dementia and cognitive impairment (STARDdem; Noel-Storr et al., 2014) can be a useful tool to improve consistency and transparency, and the application of the QUADAS 2 checklist (Whiting et al., 2011) can allow the identification of potential methodological biases, thus enabling a more effective assessment of candidate diagnostic tests. Moreover, multivariate statistical methodologies, possibly resulting in clinically-oriented tools such as nomograms, should be increasingly used to capture the complexity of the disease, both from a pathophysiological and phenotypic perspective, and to understand the actual clinical relevance of potential new biomarkers. It should be emphasized how these considerations, here paradigmatically referred to CSF, can be extended to all the candidate biomarkers for AD, regardless of their origin and nature (e.g., plasma, serum, urine, neuroimaging).

In conclusion, despite the enormous progress made in the field, there is still insufficient evidence to promote the use of candidate CSF biomarkers for AD in the routine clinical practice, As already pointed out by previous works on this topic, leaving the discussed methodological issues unaddressed raises the risk to provide clinicians with tools and tests whose answers are difficult to interpret and translate into concrete decisions. This might ultimately result in potential harm to patients, families, and healthcare systems.

## Author Contributions

MCa: study conception and writing of the manuscript. IB, GG, EL, MM and FM: literature search and drafting of the manuscript. NV and MCe: study conception and revision of the manuscript for important intellectual content.

## Conflict of Interest

MCa is supported by a research grant of the Italian Ministry of Health (GR-2016-02364975) for the project “Dementia in immigrants and ethnic minorities living in Italy clinical-epidemiological aspects and public health perspectives” (ImmiDem). MCe has received honoraria for presentations at scientific meetings and/or research funding from Nestlé and Pfizer. He is involved in the coordination of an Innovative Medicines Initiative-funded project [including partners from the European Federation Pharmaceutical Industries and Associates (Sanofi, Novartis, Servier, GSK, Lilly)]. The remaining authors declare that the research was conducted in the absence of any commercial or financial relationships that could be construed as a potential conflict of interest. The funders were not involved in the study design, collection, analysis, interpretation of data, the writing of this article or the decision to submit it for publication.
